# Intravital spectral imaging as a tool for accurate measurement of vascularization in mice

**DOI:** 10.1186/2040-2384-2-22

**Published:** 2010-10-25

**Authors:** Alicia Arranz, Ariadne Androulidaki, Berber Mol, Eleftheria Tsentelierou, Efstathios N Stathopoulos, Christos Tsatsanis, Jorge Ripoll

**Affiliations:** 1 Laboratory of Clinical Chemistry, School of Medicine, University of Crete, Heraklion 71003, Greece; 2 Laboratory of Clinical Pathology, School of Medicine, University of Crete, Heraklion 71003, Greece; 3 Institute for Electronic Structure and Laser, Foundation for Research and Technology-Hellas, Heraklion 71110, Greece

## Abstract

**Background:**

Quantitative determination of the development of new blood vessels is crucial for our understanding of the progression of several diseases, including cancer. However, in most cases a high throughput technique that is simple, accurate, user-independent and cost-effective for small animal imaging is not available.

**Methods:**

In this work we present a simple approach based on spectral imaging to increase the contrast between vessels and surrounding tissue, enabling accurate determination of the blood vessel area. This approach is put to test with a 4T1 breast cancer murine *in vivo *model and validated with histological and microvessel density analysis.

**Results:**

We found that one can accurately measure the vascularization area by using excitation/emission filter pairs which enhance the surrounding tissue's autofluorescence, significantly increasing the contrast between surrounding tissue and blood vessels. Additionally, we found excellent correlation between this technique and histological and microvessel density analysis.

**Conclusions:**

Making use of spectral imaging techniques we have shown that it is possible to accurately determine blood vessel volume intra-vitally. We believe that due to the low cost, accuracy, user-independence and simplicity of this technique, it will be of great value in those cases where *in vivo *quantitative information is necessary.

## Background

The development of new blood vessels or neoangiogenesis is a hallmark process in several biological stages but also in the progression of numerous diseases, including cancer [1]. It is known that in healthy adults angiogenesis occurs mainly during wound healing and the female reproductive cycle [2], in which case its regulation is strictly held by the balance of angiogenic activators and inhibitors. However, during tumor development this balance is disrupted and inclined towards the pro-angiogenic side: this ensures blood supply to the tumor cells and contributes to the transport of malignant cells through blood and/or lymph vessels for the development of distant metastasis [3]. It is due to this change in balance that the development of anti-angiogenic treatments as a therapeutic target in oncology has raised great interest [4]. Taking this into consideration, experimental methods to estimate tissue vascularization are crucial for the observation of blood vessels changes in the course of *in vivo *models, as well as the development of potential treatments.

Currently, optical methods exist that can provide information on oxygen saturation and blood volume *in vivo *in the intact animal [5] and functional optical spectroscopy has also been successfully applied to humans [6-9]. These techniques provide very important information and can be directly applied in a clinical environment and thus are extremely valuable. However, they suffer from low spatial resolution (>1 mm in the best of cases) as might be needed in small animal imaging studies. Other non-optical methodologies employ significantly more expensive techniques such as positron emission tomography (PET), dynamic contrast-enhanced magnetic resonance imaging (DCE-MRI), perfusion computed tomography (CT), and ultrasound (US) (see Ref. [10] for a review on the subject). However, the availability of these systems is limited and they are therefore not suitable for studies where large numbers need to be analyzed. For this purpose, *ex vivo*, histological analysis of sections with immunohistochemical staining of endothelial cell markers is probably the most recurrent method used. Nevertheless, the appearance of blood vessels in these sections is greatly influenced by their thickness and it is restricted on a small part of the tissue, limiting the accuracy of the method [10].

In order to obtain measurements as accurate as possible *in vivo *in a simple and efficient manner, we studied the potential of intravital spectral imaging for vascularization measurements. Our results demonstrate that the choice of the proper pair of exctitation/emission wavelengths allows an accurate discrimination between blood vessels and the surrounding tissues. This, together with a user-friendly software developed in-house, makes possible the quantitative determination of the area occupied by blood vessels per squared millimeter of tissue. In this work we put forward the experimental setup and approaches used, finally presenting a validation of our approach in a 4T1 breast cancer *in vivo *model by comparing with a more established technique such as microvessel density of histological sections.

## Methods

### Animals

Balb/c mice were purchased from the Hellenic Pasteur Institute (Athens, Greece) and were housed at the University of Crete School of Medicine, Greece. All procedures described below were approved by the Animal Care Committee of the University of Crete School of Medicine, Heraklion, Greece, and by the Veterinary Department of the Heraklion Prefecture, Heraklion, Greece.

### In vivo model of breast cancer cell

The mouse mammary tumor cell line 4T1 was cultured and then used for the development of the *in vivo *model. Cell culture took place in Dulbecco's Modified Eagle Medium (DMEM) medium supplemented with 10% heat-inactivated fetal bovine serum (FBS) and 1% penicillin/streptomycin (all purchased from GIBCO) at 37°C in a 5% CO_2 _humidified atmosphere.

To develop the *in vivo *model, one million 4T1 cells were implanted in the mammary fat pad of Balb/c mice [11]. A control group was subjected to the same surgical procedure, without the injection of tumor cells. The tumors were let to grow for 6 weeks at which point the mammary glands were visualized intravitally to determine the extent of neoangiogenesis following the procedure described above. Once finalized the image acquisition process, samples were collected from the different groups and histological analysis was performed.

### Histological analysis and microvessel density measurement

Mammary pad samples were surgically removed and fixed in formalin. Sections were stained with Haematoxylin-Eosin using standard techniques. For determination of microvessel density (MVD) immunohistochemical staining to detect CD31 expression was performed. Tissue sections were deparaffinized, rehydrated and then heated in a microwave oven at 600 W for 30 min in Target Retrieval Buffer, pH = 6.0 (DakoCytomation). After cooling for 20 min, standard immunohistochemistry procedures were performed using rabbit anti-mouse CD31 (dilution 1:100, Acris Antibodies, Germany) and the UltraVision Quanto Detection System HRP DAB kit (Thermo Scientific, CA, USA), following the manufacturer's recommendations.

In each case, 3-6 optical fields × 200 were selected. Each positive endothelial cell cluster of immunoreactivity within the selected field was counted as an individual vessel in addition to the morphologically identifiable vessels with a lumen.

### Statistical analysis

Comparison between groups was made using the Student's *t*-test and ANOVA test, and *p *< 0.05 was considered significant.

## Results

### Imaging Setup and Measurements

**Figure 1:**
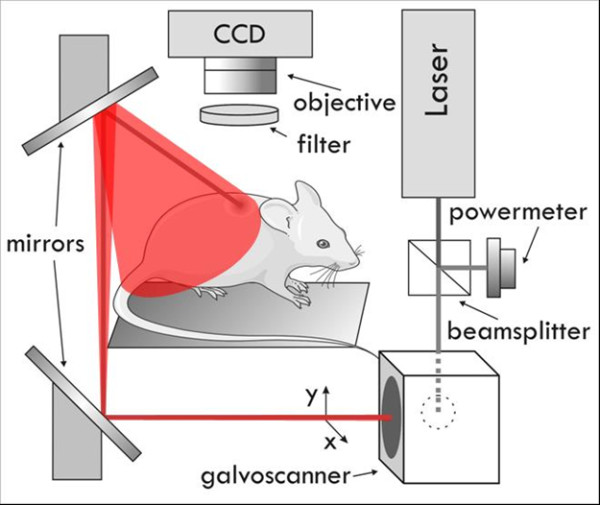
**Experimental Setup**.

**Figure 2:**
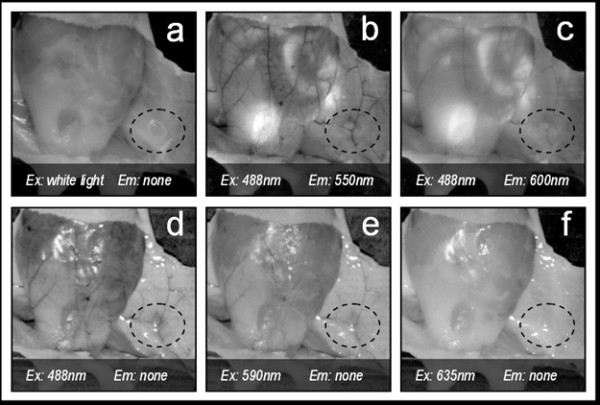
**Contrast on mammary glands (dotted line) depending on the excitation and emission wavelengths pair selected**. In this figure 'none' refers to not using an emission filter (i.e. only excitation light is collected).

A Fluorescence Molecular Tomography (FMT) setup developed at FORTH [12] has been adapted to perform intravital measurements on small animals (see Figure 1). The setup consists of several laser sources with wavelengths (488 nm, 590 nm, and 635 nm) that are guided by mirrors and scanned in reflection mode on the whole surface of the animal. During the measurements Balb/c mice where anesthetized using vaporized 1.5% Isoflurane (Tec-3, LUMIC International, Baltimore USA), and the mammary fat pads were selected as the area of interest (outlined in Figure 2). The laser is scanned while the CCD camera is open, varying the exposure time and laser speed to obtain optimal signal to noise ratios. For each excitation wavelength several emission filters are measured, building a library of emission-excitation images. These images are then used to provide the optimal source of contrast to distinguish blood vessels from surrounding tissue. A software developed in-house was designed to vary the contribution of each excitation/emission image and apply a threshold, which was later used to measure, with pixel-size accuracy, the vascularization area. Measurements on each animal took approximately 10 minutes, after which all animals were sacrificed.

### Selection of the optimal contrast

**Figure 3:**
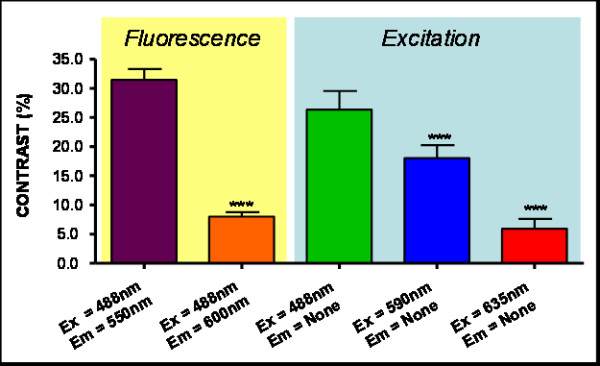
**Contrast (%)**. Results for different Excitation and Emissions. (***) indicates P < 0.001 Anova analysis.

**Figure 4:**
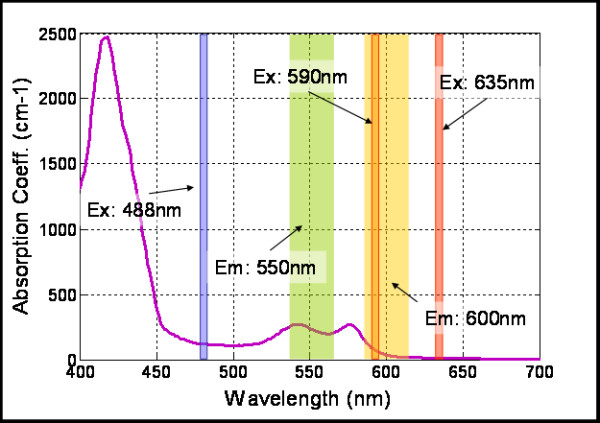
**Typical values for blood absorption curve (shown in black)**. The Laser lines and filter widths are represented for reference.

In order to select the optimal excitation/emission pair, we have studied different combinations of spectral (emission-excitation) measurements in the visible range, each one enhancing the contrast of blood and surrounding tissue differently (see Figure 2, were example images are shown). A comparison of these contrasts is shown in Figure 3, both making use of autofluorescence measurements and excitation only measurements ('none' in Figures 2 and 3 stands for no emission filter used). The reason for the changes in contrast can be understood by comparing the excitation and emission wavelengths with the blood absorption dependence with wavelength (Figure 4). As can be seen in this figure, it is the 488 nm and lower wavelengths that will present a higher contrast due to the high absorption coefficient of blood at these wavelengths. Due to the similar reasoning, the 635 nm excitation presents no contrast (in the order of 5% as shown in Figure 3).

### Software

**Figure 5:**
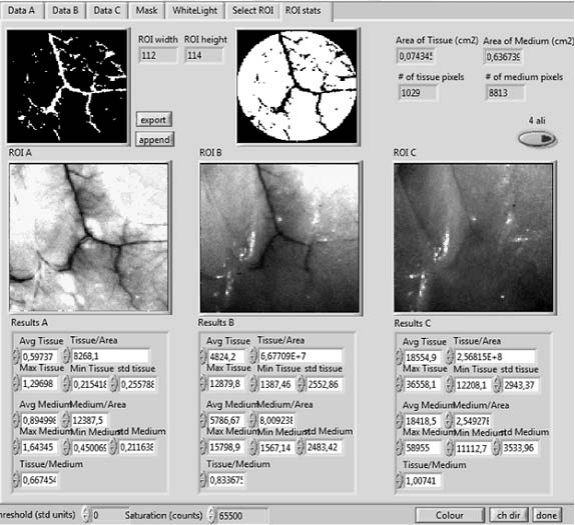
**An example of the user friendly software for measuring the vascularization area**. Regions of Interest (ROI) A, B, and C, correspond to three different excitation/emission pairs. The final mask used to measure the number of pixels corresponding to vessels or tissue can be seen at the top-left and top-middle, respectively.

**Figure 6:**
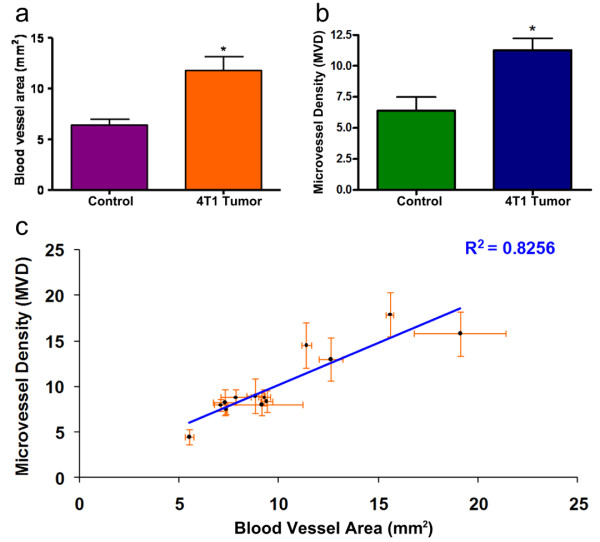
**Vascularization Area Results**. Quantification of the area of vascularization as measured with the methodology described herein, with P = 0.040 (Fig. 6a) and using Microvessel Density analysis, with P = 0.037 (Fig. 7b). (*) indicates P < 0.05 T-student analysis. Fig 6c shows the linear relationship between the vascularization area results and microvessel density analysis (R^2 ^= 0.8256).

Both the image acquisition and the image analysis software have been developed under the Labview^® ^environment. Within the image analysis window, by selecting the appropriate excitation/emission pair (Ex = 488 nm/Em = 550 nm) we are able to enhance the contrast between blood and surrounding tissue. Figure 5 shows a typical window from the in-house developed user-friendly software, showing measurements equivalent to those in Figure 2(a), (b) and 2(c). The high contrast obtained with this excitation/emission pair allowed the correct selection of the mask (see Figure 5). This mask was then used to generate the blood vessel area data shown in Figure 6. In order to obtain an accurate mask, an edge detection algorithm was used.

### Validation of the method on an in vivo breast cancer model

**Figure 7:**
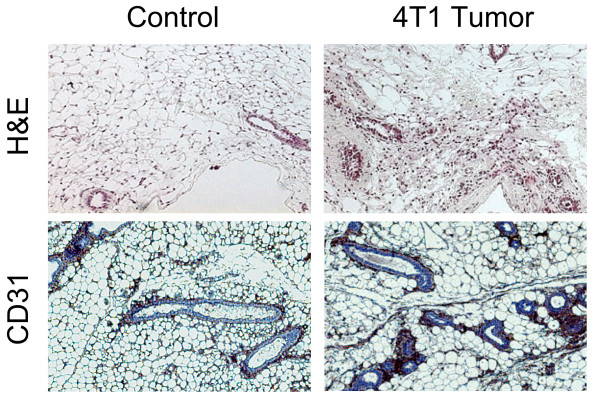
**Histological analysis**. Representative images of the histological analysis are shown. Higher presence of vasculature is appreciated in the tumor bearing sample compared with the control.

In order to validate the applicability of this method, we quantitatively measured the area of vascularization in an *in vivo *breast cancer model [11]. For this purpose, 10^6 ^cells of the breast cancer cell line 4T1 were implanted in the mammary fat pad of 13 Balb/c mice. Control mice (N = 3) were subjected to the same surgical procedure, but without the injection of tumor cells. Animals were checked on a daily basis to ensure their welfare. 6 weeks after, we measured the area of vascularization in both mammary fat pads of each mouse, following the procedure described above. In order to validate the results obtained with our method, tumor samples from the mammary fat pad were collected and histological and microvessel density (MVD) analyses performed. Results from our method are shown in Figure 6a where, as expected, tumor bearing mice show larger vascularization area. Results obtained using MVD are shown for comparison in Figure 6b. Additionally, Figure 6c shows the linear relationship between microvessel density and the vascularization areas we obtained, reflecting the high correlation between our *in vivo *method and histology. Typical histological samples, both for the H&E and CD31 staining are shown in Figure 7. These analyses corroborate the enhanced angiogenesis in tumor bearing samples.

## Discussion

The great interest in the study of angiogenesis as an essential process involved in tumorogenesis, as well as in other physiological events like wound healing, makes necessary the development of accurate methods capable of measuring changes in vascularization.

Current *in vivo *optical methods such as functional optical spectroscopy are not appropriate for small animal imaging studies due to their low spatial resolution. On the other hand, more traditional *in vivo *imaging modalities such as PET, MRI, CT and US require expensive infrastructure, and typically have a limited availability. In order to obtain higher resolution at the cost of obtaining *ex vivo *information one could make use of other traditional methods such as Immunohistochemical staining. However, Immunohistochemical staining is a time-consuming method, which requires the fixation and process of the sample, not allowing their use for other kind of analysis.

Herein we describe a simple, fast and cost-efficient method to measure the vascularization area of exposed tissue with pixel size resolution (in the order of 0.001 cm^2 ^in our case). Any technique capable of producing images that clearly represent the blood vessels present would be very useful in vascularization studies. In this paper, we explore the approach of using the high tissue autofluorescence and high blood absorption to obtain such an image, in which case blood vessels appear very dark surrounded by a bright (auto-fluorescent) background. We have shown that it is possible to accurately determine the blood vessel area of exposed tissue by using the appropriate combination of excitation/emission filter pairs, due to the enhanced contrast between the blood vessels and the surrounding autofluorescent tissue. Even though this technique has been applied here to the specific case of mammary fat pads, it can be used in any region where the blood vessels are accessible, such as the skin, the intestinal epithelium, solid tumors and other tissues. Even though this technique only probes superficial tissue, it has the advantage of being user-independent and can allow scanning of the entire tissue studied. This approach can also be extended to the analysis of human surgically removed specimen such as tumors, since it allows to accurately analyze the entire sample without any processing. Additionally, considering the simplicity of our experimental setup and the methodology described here, it should be in principle possible to incorporate other optical techniques such as laser Doppler flowmetry [13], or laser speckle imaging [14] which would enable quantitative measurement of spatio-temporal dynamics of blood flow. One drawback of these techniques when used for imaging large tissue sections is that they render grainy images due to the difficult task of obtaining a significant number of spatially different speckle patterns. The combination of the methodology presented here with laser Doppler flowmetry or speckle imaging would alleviate this fact rendering, in principle, high resolution images with dynamic information.

## Conclusions

We have presented a study of the effect that different combination of appropriate excitation/emission pairs have on enhancing the contrast of hemoglobin against surrounding tissue, finding that by selecting an excitation wavelength in the 488 nm range and an emission in the 550 nm range we obtain the maximum contrast. This enhanced contrast has enabled the measurement of vascularization area with pixel-size resolution. We have presented an imaging setup and software which enables such measurements, yielding a technique which is simple and fast, involving commercial laser sources. We believe this approach will serve as an accurate tool in biological studies where measurements of changes in vascularization over large sample populations are crucial. Additionally, due to the characteristics of the experimental setup it can be combined with other optical imaging approaches to offer quantitative information of blood flow.

## Competing interests

The authors declare that they have no competing interests.

## Authors' contributions

A. Arranz participated in the *in vivo *model, performed the measurements, and participated in the analysis and in the draft of the manuscript. BM and A. Androulidaki participated in the culture of 4T1 cells and the in vivo model. ENS and ET were involved in the histological and microvessel density analysis of the samples. CT took part in the design of the study, its coordination, the analysis and the draft of the manuscript. JR conceived the study, developed the set up and the software, and participated in the analysis and in the draft of the manuscript. All authors read and approved the final manuscript.
